# The Importance of External Contacts in Job Performance: A Study in Healthcare Organizations Using Social Network Analysis

**DOI:** 10.3390/ijerph15071345

**Published:** 2018-06-27

**Authors:** Pilar Marqués-Sánchez, María F. Muñoz-Doyague, Yolanda V. Martínez, Martin Everett, Nestor Serrano-Fuentes, Peter Van Bogaert, Ivaylo Vassilev, David Reeves

**Affiliations:** 1 SALBIS Research Group, Faculty of Health Sciences, University of Leon, Campus of Ponferrada s/n, 24401 Ponferrada, León, Spain; 2 School of Economics and Management, University of Leon, Facultad de Económicas y Empresariales, Campus de Vegazana, 24071 León, Spain; s.munoz@unileon.es; 3 NIHR School for Primary Care Research, Centre for Primary Care, University of Manchester, Oxford Road, Manchester M13 9PL, UK; yolanda.martinez@manchester.ac.uk (Y.V.M.); david.reeves@manchester.ac.uk (D.R.); 4 School of Social Sciences, University of Manchester, Oxford Road, Manchester M13 9PL, UK; martin.everett@manchester.ac.uk; 5 Faculty of Health Sciences, University of Southampton, University Road, Southampton SO17 1BJ, UK; n.serrano-fuentes@soton.ac.uk (N.S.-F.); i.i.vassilev@soton.ac.uk (I.V.); 6 Division of Nursing and Midwifery Sciences, Centre for Research and Innovation in Care (CRIC), Faculty of Medicine and Health Sciences, University of Antwerp, Universiteitsplein 1, B-2610 Wilrijk, Belgium; peter.vanbogaert@uantwerpen.be

**Keywords:** healthcare providers, job performance, social network analysis, relationships

## Abstract

There is evidence that relations between physicians and nurses within healthcare institutions might be shaped by informal aspects of such relations and by links to people external to the organization, with an impact on work performance. Social network analysis is underutilized in exploring such associations. The paper aims to describe physicians’ and nurses’ relationships outside their clinical units and to explore what kind of ties are related to job performance. A network analysis was performed on cross-sectional data. The study population consisted of 196 healthcare employees working in a public hospital and a primary healthcare centre in Spain. Relational data were analysed using the UCINET software package. Measures included: (i) sample characteristics; (ii) social network variables; and (iii) team performance ratings. Descriptive statistics (means, medians, percentages) were used to characterize staff and performance ratings. A correlational analysis was conducted to examine the strength of relationships between four different types of ties. Our findings suggest that external ties only contribute to improving the performance of physicians at both the individual and team level. They are focused on the decision-making process about the therapeutic plan and, therefore, might need to seek advice outside the workplace. In contrast, external ties are not relevant for the work performance of nurses, as they need to find solutions to immediate problems in a short period of time, having strong ties in the workplace. Social network analysis can illuminate relations within healthcare organizations and inform the development of innovative interventions.

## 1. Introduction

There is evidence that the structure of networks at the workplace can have an impact on work performance [1]. This may be through engagements within the group where individual members can use the knowledge of other members of the group (intra-group relations) [2], but can also improve performance through accessing actors, skills and knowledge external to the organization. Previous studies have indicated that innovative ideas emerge from the intersection of social worlds [3], that group effectiveness is related to close relationships and bridging relationships [4] and is also relevant for collaboration among groups of individuals in health policy processes [5]. In this sense, it has been demonstrated that the frequency of exchanges of relational resources is positively associated with satisfaction, emotions and relational cohesion [6]. Additionally, collaborative work could start from the workplace when employees contact local authorities, charities, private business and key public personnel, among others [5], exchanging experiences, knowledge and creating emerging ties to increase their social capital by bridging from one group to another [7], all of them important in the policy-making processes [8].

In the context of healthcare, there is evidence that relationships between healthcare professionals might be optimized through access to advice or help at work, by enhancing collective efficacy [9] and by building positive relations between different groups, organisational units and hierarchical levels [10,11,12]. For example, Mehra et al. [13] showed that the embeddedness of leaders in the friendship network of their subordinates and supervisors had implications for group performance and leader reputation. Furthermore, healthcare professionals build friendship networks with employees of the same role, creating a platform for the effective spread of information when there is an absence of clinical leaders [14]. This might be through the engagement of employees [15], developing effective relationships over formal hierarchical positions [5] and building a collaborative organizational culture [16]. Network analysis seems appropriate in the context of health policies, as it offers possibilities to represent network processes and explore the bridging role played by opinion leaders and other actors in the network [17]. This perspective, through the social network analysis method, could facilitate systemic thinking capable of addressing the challenges and uncertainty related to a sustainable model of medical care. The analysis of structural patterns and associated factors can provide a set of indicators that are not available to other methodologies [18].

However, barriers between professional groups tend to inhibit inter-professional interaction partners [19], displaying different structural configurations and, therefore, influencing job performance and atmosphere within the team. The importance of ties across hierarchies, with other departments inside organizations or other individuals outside an organization to improve performance, has been reported previously by Krackhardt Stern [20]. Meltzer et al. [21] demonstrated that employees in health organizations were connected with other peers to get advice, help in their task or to find some emotional support. Drawing on existing institutional processes, as well as the effective use of new Information and Communication Technology (ICT) can all be mobilized in enhancing effective networks within and across teams and organizations that can be geographically dispersed [22].

Despite the importance of social network research in organizations [23], there are few studies looking specifically at the effectiveness of social networks in healthcare settings and their contributions to the quality of patient care [24]. There is a gap in the literature regarding the relationship between social networks (including ties within and outside healthcare organization) and work performance in healthcare settings. This paper aims to describe professional relationships of healthcare professionals inside and outside their healthcare organization and to explore the types of advice-seeking ties related to job performance. The results obtained can inform the decision making process and support the work of managers of health organizations. The study also aims to contribute to the better understanding of relations between people working within the healthcare system and offer inroads to understanding the processes that can shape performance.

The following sections describe the operationalisation of these objectives, the instruments used and the process for data collection, presentation and interpretation of the results, as well as a summary of the key points.

## 2. Materials and Methods

### 2.1. Study Design

The study was conducted between 1 June and 30 October 2008 in a public hospital (Hospital El Bierzo) and a primary care health centre (Bembibre) in Spain. These organizations were selected according to geographical proximity and the acceptance by the managers to be part of the research. One hundred ninety six healthcare professionals (physicians, nurses, healthcare assistants and laboratory technicians) participated in the study. The teams included in this study were: Surgical Unit, Dialysis Unit, Management Team, General Medicine, Microbiology Laboratory, Paediatric Unit and Intensive Care (all of these teams at the hospital); and Primary Care (at the health centre). This reflected the key role that occupational characteristics were expected to have in shaping the structure of the networks of health professionals [25].

### 2.2. Ethics

The protocol was presented to the hospital managers before data collection in order to obtain ethical approval. Ethics approval was given by the Economics Department (University of León), by the manager of the Hospital Bierzo, the manager of the Bembibre primary care health centre and the Ethics Committee of the health area Bierzo.

### 2.3. Variables and Data Collection Instrument

The questionnaire was designed specifically for the purposes of this study and adapted from West et al. [25], Sparrowe et al. [26], Johnson [27], Barrick et al. [28] and Griffin et al. [29]. It included four items to measure social networks and three measures of performance and other characteristics of the sample. Social network data and performance data evaluated by employees and managers were collected based on a Likert scale and closed items, as shown in Table 1 and Table 2.

#### 2.3.1. Characteristics of the Sample

Socio-demographic data about respondents included gender (male/female), job role (physician, nurse, healthcare assistant and laboratory technicians), time in the job (0–5 years, 6–10 years, 11–20 years, 21–30 years, 31 or more years) and teams.

Structural social network characteristics were used as independent variables. We examined advice networks relating to internal and external ties. Numbers of internal and external ties are forms of out-degree measure, providing an indication of a person’s (or “node’s”) centrality or location in the network. The out-degree describes the number of connections from one node to other nodes in the network and therefore represents the node’s degree of influence [30]. If a node has a high number of out-degrees, this means that many connections start from him/her and, therefore, that he/she is able to reach other adjacent individuals, groups or organizations. Thus, internal ties were used to measure advice networks inside the organization. Each employee was asked to name individuals in other departments within the organization (outside of his/her own team) with whom he/she had established contact to ask for advice to improve his/her working life at his/her workplace. The total number of co-workers named by an employee represented his/her number of internal ties. In contrast, external ties were used to measure advice networks outside the organization. Each individual was asked to indicate if he/she had or not contact with selected groups of interest (family, friends, professional institutions, trade unions, the University, governmental institutions, hospitals, primary care health centres and other medical establishments) to improve their work situation (asking for advice to improve their working life) or workplace (asking for advice to improve their working environment). The total number of external groups with whom an employee had contacts was taken as the measure of his/her external advice network. All the items are specified in Table 1.

The job performance was identified as a dependent variable. Data were collected at three different levels within the organizations: (i) member performance ratings of each other, (ii) supervisory staff ratings of team performance and (iii) senior manager ratings of team performance. Member performance ratings of each other were adapted from Sparrowe et al. [26], and each employee was asked to rate the other members of their team on this item using a 5-point Likert scale from 1 (strongly disagree) to 5 (strongly agree). The job performance score for each staff member was computed as the average of their ratings from all other members of their team. Missing ratings were replaced by the average across the ratings that the person received. In this way, we were also able to compute scores for the 58 staff members who did not provide any performance ratings of their team members. Regarding supervisory staff ratings of team performance (Table 2), the job performance evaluation was developed including initiative [29], proactive attitude [26], time management [28], sociability and environment adaptation [27] and punctuality [29], among others. Supervisors evaluated their team with a 5-point Likert scale from 1 (never) to 5 (always) for each item. Physicians and nurses were rated by their supervisors. The final score for each team was the mean rating across all items on the questionnaire. Concerning senior manager ratings of team performance, the senior managers provided an evaluation of each team after studying the team’s performance ratio scores. Performance ratios were quantitative indicators based on organization records and produced by the departments of financial management, quality, training and the managers of nurses and physicians. Each team had specific indicators, and there were other common indicators for all teams, as can be seen in Table 2. The senior managers evaluated each team using a 10-point scale from 1 (very low) to 10 (excellent).

#### 2.3.2. Analysis of Staff Job Performance Ratings

Univariate and multivariate regression analyses were used to investigate relationships between job performance ratings and the numbers of each type of tie while controlling for team and staff characteristics. First, a series of univariate regressions was run to examine the relationship of job performance to each type of tie and to each of the other explanatory variables in turn (team, staff gender, staff job role, time in job). Next, a series of multivariate regressions was conducted between job performance and each type of tie, controlling in each case for team, gender, job role and time in job. This analysis also examined if the relationship between the ties and job performance differed between types of staff, by including an interaction term between the number of ties and job role. A final multivariate analysis included all the types found to be significantly associated with job performance in the individual multivariate analyses, to determine if they remained significant once controlled for one another.

#### 2.3.3. Analysis of Senior Manager and Supervisor Ratings of Team Performance

With only eight teams, analysis that could be performed on the senior manager and supervisor ratings was quite limited. We restricted this to examination of Spearman non-parametric correlations and scatter plots between ratings and numbers of ties (at the team level) for each type of tie. The UCINET software was used for the analysis of the relational data [31].

### 2.4. Statistical Analysis

Descriptive statistics (means, medians, percentages) were used to characterize the features of the staff and the job performance ratings and correlation analysis to examine the strength of relationships between the four different types of ties.

## 3. Results

### 3.1. Characteristics of the Sample

Table 3 shows characteristics of both respondents and non-respondents to the questionnaire. The gender breakdown was similar in both groups with almost three-quarters being female. Regarding the job role, non-respondents (compared to respondents) were more likely to be physicians. The differences in gender by job role were significant (Chi-squared 49.2, *p*-value < 0.001).

Table 4 gives descriptive statistics on the numbers of each type of tie and job performance scores broken down by job role (physician versus nursing employees).

**Figure 1:**
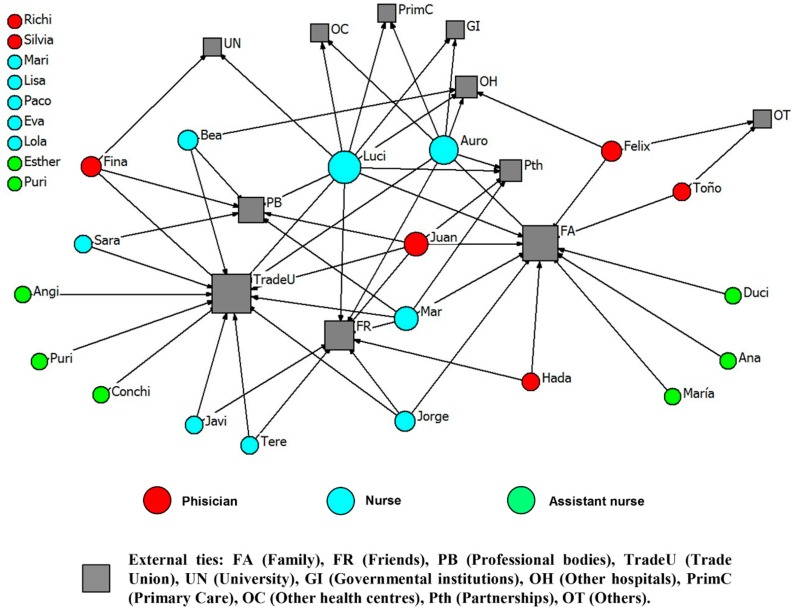
Advice network for General Medicine. External ties to improve working life (names are fictitious).

**Figure 2:**
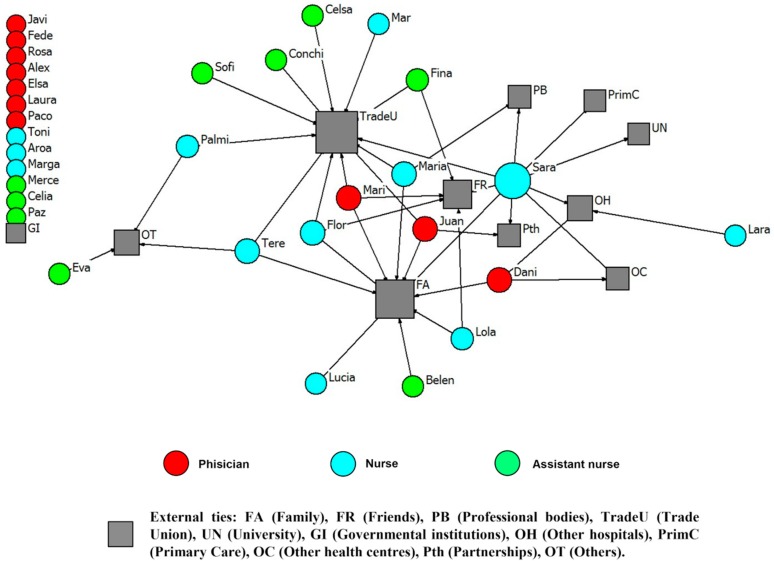
Advice network for the Surgery Unit. External ties to improve workplace (names are fictitious).

**Figure 3:**
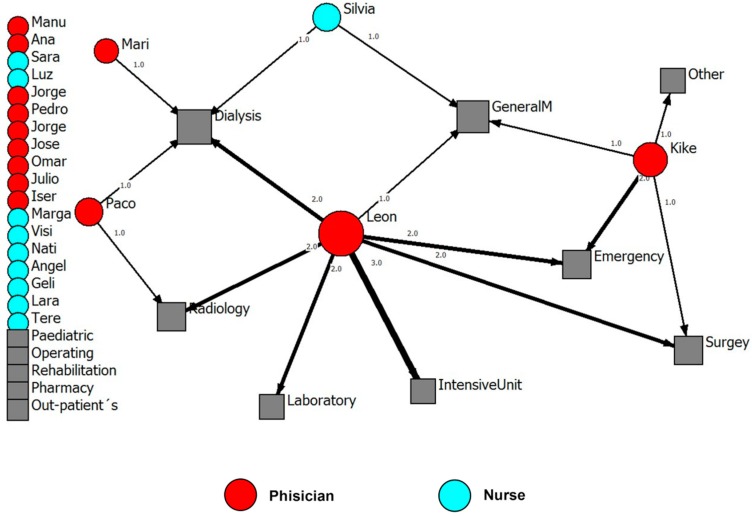
Advice network for the management team. Internal ties to improve working life (names are fictitious). Note: The width of the lines shows the number of contacts that each employee has with the corresponding department. The number of contacts is written next to each line. Hospital departments on the left side (grey) are departments that were not mentioned by respondents.

**Figure 4:**
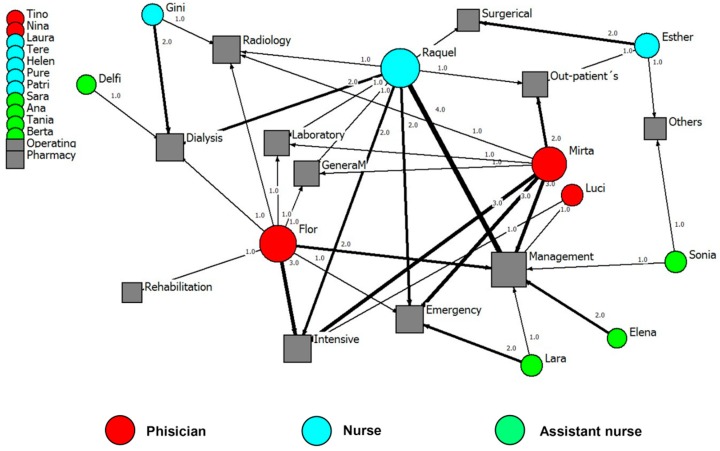
Advice network for the Paediatric Unit. Internal ties to improve workplace (names are fictitious). Note: The width of the lines shows the number of contacts that each employee has with the corresponding department. The number of contacts is written next to each line. Hospital departments on the left side (grey) are departments that were not mentioned by respondents.

Physicians reported higher mean numbers of all types of ties, compared to nursing employees (an example of each type of tie is presented). Social relationships of health professionals with other external ties (e.g., family, professional bodies, the University) in order to get advice and improve working life are represented in Figure 1, Figure 2, Figure 3 and Figure 4.

However, the mean values were inflated by a small number of staff with many numbers of ties, and the median numbers of ties are lower in all cases, with significant group differences on external ties to improve the workplace (median 2.5 vs. 2.0, *p*-value < 0.05) and internal ties to improve the workplace (median 2.0 vs. 0, respectively, *p*-value < 0.01) only. Mean job performance scores were also significantly different, though higher for nursing employees (mean 3.48 vs. 3.78, *p*-value < 0.001).

**Figure 5:**
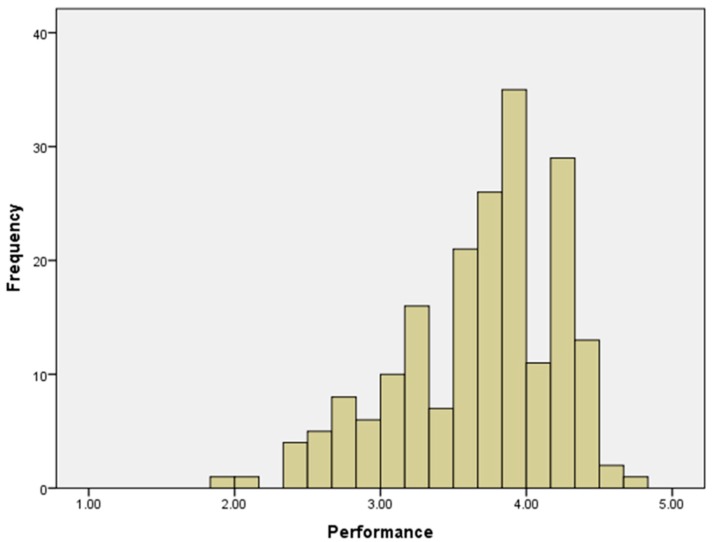
Histogram of performance scores for individual staff members.

Table 5 shows correlations between different types of ties. There were high correlations between the two kinds of external ties (to improve working life and to improve the workplace) for both physicians (*r* = 0.74, *p*-value < 0.01) and nursing employees (*r* = 0.79, *p*-value < 0.01). Nevertheless, relationships between numbers of the corresponding two kinds of internal ties and external and internal ties were weaker. The only significant relationships were between internal ties to improve the workplace and external ties for the same purpose (*r* = 0.24, *p*-value < 0.05) and with internal ties to improve the working life (*r* = 0.38, *p*-value < 0.01). Figure 5 illustrates the distribution of the individual staff job performance scores, rated by their fellow team members with an overall mean score of 3.6 (SD 0.5).

### 3.2. Predictors of Staff-Level Job Performance Ratings

In Table 6, the univariate regressions showed that some staff characteristics were significantly related to the job performance ratings of participants by fellow members. Being a member of the Dialysis, Microbiology Laboratory or Paediatric team was associated with mean increases in performance rating of 0.22, 0.18 and 0.08 points, respectively, compared to participants in the Intensive Care team, whereas the managerial team received the lowest rating overall (−0.93 compared to Intensive Care) (omnibus *p*-value < 0.001). Team membership accounted for 31% of all the variation in job performance ratings. Being a female was significantly associated with a 0.40 increase (*p* < 0.001). Being a nurse was associated with a 0.30 increase compared to being a physician (*p* < 0.001). Being 21 years old or more in the job was associated with a 0.35 point decrease in job performance rating compared to having ten years or less in the job (omnibus *p*-value < 0.001). However, job performance ratings were not significantly related in these univariate analyses to any of the four types of ties (*p* > 0.05).

In the multivariate analysis (Table 7), examining each form of tie separately, external and internal ties to improve the workplace were significantly associated with a 0.10 and 0.02 increase respectively in mean job performance ratings for each additional tie (*p* < 0.001 and *p* = 0.044), after controlling for team and member characteristics. An interaction between external ties to improve the workplace and job role was significantly associated with a 0.11 decrease in the overall mean job performance ratings (*p* = 0.003). External and internal ties to improve the working life were not significantly associated with job performance ratings.

Table 8 shows the multivariate analysis of job performance ratings including all significant ties from Table 7 and controlling for team and member characteristics. Relative to the overall mean job performance rating of 3.6 (Figure 5), the Dialysis and Microbiology Laboratory teams were associated with mean increases in the rating of 0.18 and 0.07 points, respectively, compared to participants in the Intensive Care team (omnibus *p*-value < 0.001). Nurse role was associated with a 0.32 increase compared to being a physician (*p* = 0.006). External ties to improve the workplace remained significantly associated with a 0.10 increase in the overall mean job performance ratings (*p* = 0.005). An interaction between external ties to improve workplace and job role remained significantly associated with a 0.12 decrease in the overall mean job performance ratings (*p* = 0.004). Internal ties to improve the workplace did not remain significant in this model. This model accounted for 43% of all the variation in job performance ratings.

**Figure 6:**
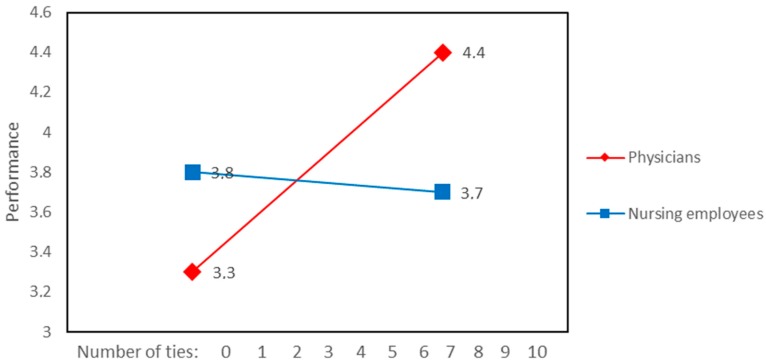
Interaction between external ties to improve the workplace and job role associated with performance rated by team members.

**Figure 7:**
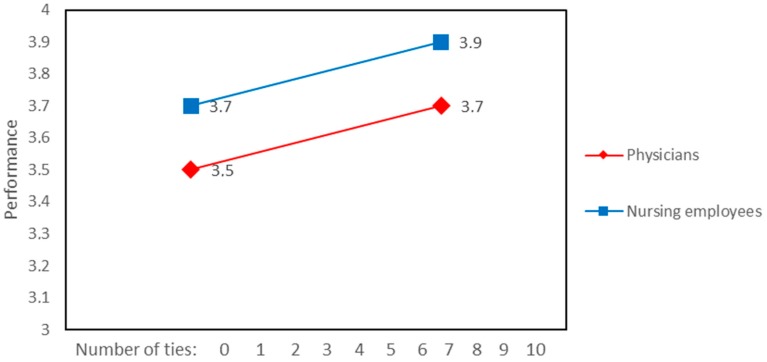
Internal ties to improve workplace and job role associated with performance rated by team members.

A significant interaction was found between external ties to improve the workplace and job role in the prediction of job performance rated by team members (Figure 6). This interaction shows that job performance rated by team members was higher for physicians with more external ties to improve workplace in comparison with nurses. Figure 7 illustrates the significant association between job performance rated by team members and job role. This relationship was not affected by the number of internal ties to improve the workplace.

### 3.3. Team Level Results

**Figure 8:**
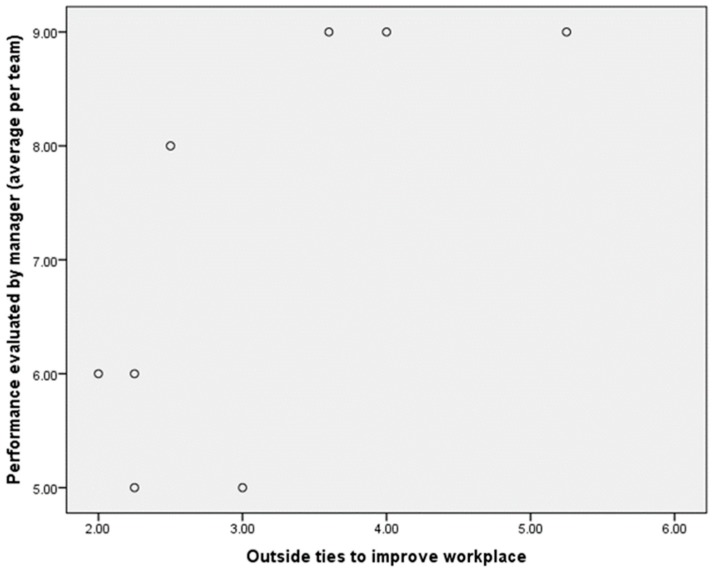
Scattergram at team level for physicians between performance evaluated by manager and external ties outside organization to improve the workplace.

Table 9 shows correlations between all the different types of ties and job performance evaluated by senior managers and supervisors. Most of the correlations were not significant except for external ties to improve the workplace and job performance evaluated by senior managers among physicians (*r* = 0.70, *p*-value < 0.05). Figure 8 shows a scattergram of this significant correlation.

## 4. Discussion

The aim of our study was to describe professional relationships of healthcare workers inside and outside their healthcare organisation and to explore the types of advice-seeking ties related to job performance. To address this research gap, a social network approach was used to describe how health employees’ ties build networks to transfer resources such as giving advice outside the organisation. Job performance was evaluated at the individual, supervisor and senior manager levels in order to investigate and compare social networks and group dependences on individual responses [32].

Our findings indicate that there is a link between having external contacts outside the healthcare workplace and job performance. However, there were differences between physicians and nurses. Thus, at the individual and team level, external ties to improve the workplace and job performance evaluated by senior managers were positively correlated, but only for physicians. Similar findings have been reported previously suggesting that physicians’ network density and external ties can enhance performance and “allow contact with fresh, not redundant knowledge” [33]. The explanation that physicians have more external ties might be related to seeking advice, as doctors are more focused on the decision-making process about therapeutic plans. This suggestion is in line with Chung and Jackson [34], who stated that “effective performance of knowledge intensive teams depends on free-flowing questioning, advice giving, and knowledge used among team members and across team boundaries”. The absence of statistically-significant associations between nurses’ ties and job performance might be related to role, competences and expectations from the nursing role associated with the need to develop very close relationships with team members in providing answers and developing solutions in a short period of time. This fact implies a high level of inter-personal trust in dealing with emotional stress and, thus, the need to create strong networks of people with similar experiences inside the workplace [35]. Additionally, our findings suggest that senior managers were more aware of the external connections that physicians have, in comparison to those of nurses. This might be related to homophily (the tendency of people to interact more with their own kind [2]) along gender and occupation lines (i.e., the majority of senior managers are male and physicians). Professional groups, within healthcare organisations, might also have a low level of interactions in the workplace as a consequence of conflicts. For example, communication between nurses and physicians is complex and even difficult to track sometimes, and there is a lack of communication, which has a potentially negative impact on outcomes [36]. Some researchers have highlighted that gaps between nurses and doctors can inhibit the development of inter-professional networks [33,37]. 

However, there is evidence that external connections develop knowledge translation, which is potentially relevant to innovation in health contexts [38], and they are therefore potentially beneficial for both physicians and nurses. Therefore, different ways to access a wider network and outside contacts should be considered in relation to professional development [35]. In this sense, the use of social network analysis might allow us to “dive” into these social structures of teams within organizations and potentially lead to the development of innovative interventions. For example, we suggest that in order to improve performance, senior managers and supervisors might have a key role to play by encouraging healthcare professionals to share information with individuals and teams within and across organisations and also by developing an understanding of the roles different employees play within the existing team configurations. Furthermore, the engagement of nurses in research and innovative practices might allow them to negotiate and navigate different networks in order to find new resources [39]. White et al. [40] found that in a routine context, the formal leader can encourage generalised exchange, which brings indirect reciprocity. In this sense, managers could draw on interventions that allow the identification, lining up with and development of shared strategies with key external actors. For example, the “Net-Map” technique might be applied to map all the contacts in different circles, identifying who are the closest contacts. This combines participatory diagnosis and strategic planning with the intervention of actors involved in an important public health or community development issue [41]. 

Finally, to include innovation in healthcare settings, it might be useful to consider external ties as a source of knowledge transfer. In this sense, the use of social network analysis might be relevant for identifying and building relationships with external contacts.

### Limitations

This study shows an important limitation regarding the number of participants. Furthermore, although these findings report on the interactions of a small group of healthcare providers in a region of Spain and thus may not be reproduced in other populations, they provide a starting point to consider the potential role of social networks in improving job performance in healthcare organizations. Finally, the respondents might have been concerned about confidentiality with possible impact on the truthfulness of their responses about seeking advice. 

## 5. Conclusions

This study adds evidence of healthcare professionals’ networks and their relationship to performance in healthcare organisations [4,13]. Nurse’s and doctor’s networks are different, with a different impact on their job performance. Thus, external ties are relevant to improving the job performance in physicians at both the individual and team level (evaluated by senior managers), as they are focused on the decision-making process about the therapeutic plan and the need to seek advice outside of the workplace. In contrast, external ties are not relevant for the job performance of nurses as they tend to be responsible for finding short-term solutions and in a short period of time. They have strong ties in the workplace based on a high level of trust, gossiping and emotional stress.

This study shows how network analysis offers the opportunity to carry out future lines of research on the optimization of bridges created between different institutions (e.g., hospitals and universities) potentially leading to identifying key players and facilitating a transfer of knowledge and ideas.

## Figures and Tables

**Table 1:** Social networks items. Questionnaire items, based on network questions description.

Items
Employees were asked to respond to four items related to their advice-seeking behaviour: Internal ties to improve the working life. Write down the names of your co-workers from other departments to whom you ask advice related to improving your career development. Internal ties to improve the workplace. Write down the names of your co-workers from other departments to whom you ask advice related to improving your working environment. External ties to improve working life. Have you sought advice related to improving your career development from any of the people on this list? Tick yes or no in each option.
Family
Friends
Professional institutions
Trade unions
University
Governmental institutions (city councils, autonomous governments, etc.)
Hospitals
Primary care health centre
Other medical institutions
Professional associations
Others (specified by the respondent)
External ties to improve the workplace. Have you sought advice related to improve your working environment from any of the people on this list? Tick yes or no in each option.
Family
Friends
Professional institutions
Trade unions
University
Governmental institutions (city councils, autonomous governments, etc.)
Hospitals
Primary care health centre
Other medical institutions
Professional associations
Others (specified by the respondent)

**Table 2:** Performance items. Supervisory staff ratings of team performance.

**Items**	
(1) Do they suggest new projects in their department?	
(2) Are they proactive in problem-solving?	
(3) Do they take responsibilities?	
(4) Is the team competent in fulfilling processes, procedures or protocols?	
(5) His/her communication is correct with both patients and his/her colleagues	
(6) He/she completes all forms (records, medical histories, tests, etc.) in a clear and organised way?	
(7) Does he/she perform his/her duties with the objective to be the most efficient and effective possible?	
(8) Do they evaluate their tasks?	
(9) Are they punctual in their job?	
**Senior managers rating of team performance**	
**Specific indicators (depend of the team)** Departments of financial management, quality	People with one learning activity (for continuing professional development)
	People with more than one learning activity
	Number of clinical processes
	Average bed occupancy rate
	Average bed occupancy (objective)
	Average inpatient bed occupancy (standard deviation)
	Number of inpatient falls
	Review the check-list of crash trolley items.
	Biological risks
	Average delay clinical tests
	Average delay clinical tests (objective)
	Average delay clinical tests (standard deviation)
	Number of reviewed patients
	Number of reviewed patients (objective)
	Number of reviewed patients (standard deviation)
	Proposal of health services
	Results from health services
	Results from health offered services (objective)
	Improvement projects
**Common indicators** Training and the managers of nurses and physicians	Number of employees
	Number of leaves
	Number. of trade unionist
	Number. non-respondents

**Table 3:** Characteristics of the study sample.

Characteristics		Responder		Non-Responder	
		*N* = 138	%	*N* = 58	%
Gender	Male	39	28.3%	17	29.3%
	Female	99	71.7%	41	70.7%
Job Role	Physician	39	28.3%	23	39.7%
	Nurse	62	44.9%	24	41.4%
	Healthcare assistant	33	23.9%	11	19.0%
	Laboratory technician	4	2.9%	0	0%
Time in the Job	0–5 years	21	15.2%	0	0%
	6–10 years	14	10.1%	8	13.8%
	11–20 years	46	33.3%	20	34.5%
	21–30 years	34	24.6%	18	31.0%
	31 or more years	23	16.7%	12	20.7%
Team	Primary Care	22	15.9%	16	27.6%
	Surgical Unit	22	15.9%	9	15.5%
	Dialysis Unit	12	8.7%	4	6.9%
	Management Team	16	11.6%	7	12.1%
	General Medicine	19	13.8%	9	15.5%
	Microbiology Laboratory	7	5.1%	0	0%
	Paediatric Unit	16	11.6%	5	8.6%
	Intensive Care	24	17.4%	8	13.8%

**Table 4:** Descriptive statistics for ties and performance at the individual level by job role.

Variables		*N*	Mean	Median	SD	Min	Max	*p*-Value
External ties to improve working life	Physician	42	3.19	3.00	2.18	0	8	0.179 *
	Nursing Employees	106	2.75	2.00	2.15	0	10	
External ties to improve workplace	Physician	42	2.76	2.50	1.98	0	8	0.025 *
	Nursing Employees	107	2.14	2.00	2.23	0	10	
Internal ties to improve working life	Physician	31	1.90	0	3.63	0	14	0.204 *
	Nursing Employees	92	0.79	0	2.03	0	15	
Internal ties to improve workplace	Physician	32	4.03	2.00	5.18	0	17	0.009 *
	Nursing Employees	91	1.21	0	2.06	0	15	
Performance at individual level	Physician	62	3.48	3.55	0.63	1.90	4.70	0.001 †
	Nursing Employees	134	3.78	3.88	0.49	2.00	4.56	

Note: * Mann–Whitney U; † *t*-test.

**Table 5:** Correlations for the individual level: physicians and nursing employees.

Network Variables	Physicians			Nursing Employees		
	1	2	3	1	2	3
1-External ties to improve working life						
2-External ties to improve workplace	0.739 **			0.788 **		
3-Internal ties to improve working life	−0.060	−0.219		0.060	−0.020	
4-Internal ties to improve workplace	0.231	0.256	0.284	0.134	0.244 *	0.384 **

Note: * *p* < 0.05; ** *p* < 0.01.

**Table 6:** Univariate analysis of job performance ratings by network members.

Variable		Univariate Analysis		% of Variance Explained ^1^
		Co-eff	*p*-Value	
**Team** (compared to team 1 Intensive Care)	Surgery	−0.0005	<0.001	31%
	Dialysis	0.22		
	Manager	−0.93		
	Microbiology Laboratory	0.18		
	General Medicine	−0.19		
	Paediatric	0.08		
	Primary Care	−0.31		
**External ties to improve working life**		0.01	0.31	0%
**External ties to improve workplace**		0.02	0.24	0.2%
**Internal ties to improve working life**		−0.01	0.56	0.5%
**Internal ties to improve workplace**		0.01	0.25	0.2%
**Female gender** (compared to male)		0.40	<0.001	10%
**Job role nurse** (compared to physician)		0.30	<0.001	5%
**Time in job** (compared to 0–10 years)	11–20	−0.09	<0.001	6%
	21plus	−0.35		

Note: ^1^ Adjusted R-squared.

**Table 7:** Summary of multivariate analyses of staff job performance scores: separate models using external ties to improve working life, external ties to improve workplace, internal ties to improve working life and internal ties to improve workplace.

Independent Variable		Regression Coefficient and *p*-Value ^3^	
		Co-eff	*p*-Value
**External ties to improve working life**	Main effect ^1^	0.01	0.42
	Interaction with job role	−0.02	0.46
**% of variance explained ^2^**		35%	
**External ties to improve workplace**	Main effect	0.10	0.001
	Interaction with job role	−0.11	0.003
**% of variance explained ^1^**		38%	
**Internal ties to improve working life**	Main effect ^1^	0.003	0.81
	Interaction with job role	0.009	0.76
**% of variance explained ^2^**		36%	
**Internal ties to improve workplace**	Main effect ^1^	0.02	0.044
	Interaction with job role	−0.003	0.90
**% of variance explained ^2^**		39%	

Note: ^1^ After removal of non-significant interaction term; ^2^ adjusted R-squared for the full model; ^3^ each multivariate analysis was controlled for team, gender, job role and time in job.

**Table 8:** Multivariate analysis of job performance ratings by network members combining external and internal ties to improve workplace, and controlling for team and member characteristics.

Variable		Multivariate Analysis	
		Co-eff	*p*-Value
**Team** (compared to Team 1 Intensive Care)	Surgery	−0.13	<0.001
	Dialysis	0.18	
	Manager	−0.68	
	Microbiology laboratory	0.07	
	General Medicine	−0.25	
	Paediatric	−0.11	
**External ties to improve the workplace**	Main effect	0.10	0.005
	Interaction with job role	−0.12	0.004
**Internal ties to improve the workplace**	Main effect ^1^	0.01	0.20
	Interaction with job role	0.02	0.413
**Gender female** (compared to male)		0.21	0.058
**Job role nurse** (compared to physician)		0.32	0.006
**Time in job** (compared to 0–10 years)	11–20	−0.17	0.161
	21+	−0.16	
**% of variance explained ^2^**		43%	

Note: ^1^ After removal of non-significant interaction term; ^2^ adjusted R-squared.

**Table 9:** Results of Spearman correlations between ties and job performance evaluated by senior managers and supervisors.

Items	Senior Managers Evaluation of Physicians	Senior Manager Evaluation of Nurses	Physicians’ Supervisor Evaluation	Nurses’ Supervisor Evaluation
External ties to improve the working life	0.590	−0.358	0.524	−0.342
External ties to improve the workplace	0.708 *	0.272	0.434	−0.220
Internal ties to improve the working life	0.661	−227	0.414	0.559
Internal ties to improve the workplace	−0.353	0.548	−0.091	0.252

Note: * *p* < 0.05.
